# Transperineal Versus Transrectal Prostate Biopsy in the MRI-Targeted Era: A Contemporary Narrative Review

**DOI:** 10.3390/biomedicines14081723

**Published:** 2026-07-31

**Authors:** Silviu Latcu, Flaviu Lucian Petrica, Dorin Novacescu, Roland Costica, Vlad Dema, Victor Pasecinic, Ionel Chiriac, Mihai Baghina, Radu Caprariu, Radu Dragomir, Razvan Bobora, Alin Cumpanas

**Affiliations:** 1 Bastion Policlinic, Mircea cel Batran Street, No. 122C, 300600 Timisoara, Romania; silviu.latcu@umft.ro (S.L.); medical@policlinicabastion.ro (F.L.P.); policlinicabastion@gmail.com (R.C.); vlad.dema@umft.ro (V.D.); victor.pasecinic@umft.ro (V.P.); ionel.chiriac@umft.ro (I.C.); mihai.baghina@yahoo.com (M.B.); 2 Doctoral School, Victor Babes University of Medicine and Pharmacy Timisoara, E. Murgu Square, No. 2, 300041 Timisoara, Romania; razvan.bobora@umft.ro; 3 Department XV, Discipline of Urology, Victor Babes University of Medicine and Pharmacy Timisoara, E. Murgu Square, No. 2, 300041 Timisoara, Romania; cumpanas.alin@umft.ro; 4 Department II of Microscopic Morphology, Victor Babes University of Medicine and Pharmacy Timisoara, E. Murgu Square, No. 2, 300041 Timisoara, Romania; 5 Department XV, Discipline of Radiology and Medical Imaging, Victor Babes University of Medicine and Pharmacy Timisoara, E. Murgu Square, No. 2, 300041 Timisoara, Romania; 6 Department of Oncology, Victor Babes University of Medicine and Pharmacy Timisoara, E. Murgu Square, No. 2, 300041 Timisoara, Romania; radu.dragomir@umft.ro; 7 Medical Oncology Department, Emergency Clinical County Hospital Timisoara, Victor Babeș Street, No. 22, 300595 Timisoara, Romania

**Keywords:** prostate cancer, prostate biopsy, transperineal biopsy, transrectal biopsy, multiparametric MRI, MRI-targeted biopsy, fusion biopsy, PI-RADS, clinically significant prostate cancer, biopsy complications

## Abstract

Prostate biopsy is among the most frequently performed diagnostic procedures in men, and two shifts have reshaped it over the past decade: the incorporation of multiparametric MRI (mpMRI) with targeted sampling, and migration from the transrectal to the transperineal route. Because these changes are usually adopted together, the practical choice facing many units is between a contemporary transperineal MRI-targeted pathway and the historical transrectal systematic one. This narrative review weights cancer detection, procedure-related harms, and implementation approximately equally, drawing on evidence that isolates route from targeting wherever possible. Across randomized trials and meta-analyses, the two pathways achieve broadly comparable detection of clinically significant cancer (ISUP grade group ≥ 2), although the transperineal route samples anterior and apical tumors more effectively and the largest randomized comparison reported a modest transperineal advantage. The principal transperineal benefit is a marked reduction in infectious complications, enabling reduced antibiotic prophylaxis and improved stewardship; its main cost is greater procedural discomfort. We review mpMRI and PI-RADS with illustrative examples, fusion technique, cost-effectiveness, special populations, emerging adjuncts, and divergent guidelines, concluding that route and targeting decisions are best individualized, with infection risk and lesion location dominant.

## 1. Introduction

Prostate cancer is the second most frequently diagnosed malignancy in men and a leading cause of cancer-related death worldwide. The most recent global estimates attributed approximately 1.47 million new cases and nearly 400,000 deaths to the disease in a single year, with wide international variation in both incidence and mortality [1,2]. Contemporary epidemiologic analyses emphasize that much of this variation reflects differences in detection intensity and the dissemination of prostate-specific antigen (PSA) testing rather than true differences in underlying disease, which places the accuracy and safety of the diagnostic pathway at the center of any rational approach to the disease [3]. Against this backdrop, prostate biopsy occupies a pivotal position: it is the procedure that converts a clinical suspicion—raised by an elevated PSA, an abnormal digital rectal examination, or a suspicious magnetic resonance imaging (MRI) finding—into a histological diagnosis. Prostate biopsy is one of the most frequently performed diagnostic procedures in men, which makes both its diagnostic yield and its safety profile questions of genuine population-level importance [4].

For three decades, the default approach was transrectal ultrasound (TRUS)-guided systematic biopsy, in which ten to twelve cores are taken in a standardized spatial distribution through the rectal wall [5,6]. This approach is quick, well tolerated under local anesthesia, and can be performed in the office setting, but it has two well-recognized limitations. First, a blind systematic scheme that samples predominantly the posterolateral peripheral zone systematically under-samples the anterior gland, the apex, and the transition zone, where a clinically meaningful minority of significant tumors arise [7,8]. Second, passing the biopsy needle through the rectal mucosa inoculates the prostatic and bloodstream compartments with enteric organisms, producing a non-trivial rate of infectious complications that has risen over time with the global increase in fluoroquinolone-resistant rectal flora [9].

Two distinct innovations emerged to address these limitations, and although they are conceptually separable, they have become tightly intertwined in modern practice. The first is the use of multiparametric MRI (mpMRI) before biopsy to identify and characterize suspicious lesions, combined with targeted sampling of those lesions—either by cognitive registration, by software-assisted fusion of MRI with real-time ultrasound, or by direct in-bore guidance. The second is a return to the transperineal access route, in which the needle traverses the perineal skin rather than the rectum, thereby avoiding direct fecal contamination of the needle tract and providing a more natural cranio–caudal trajectory to the anterior and apical prostate. Modern freehand local-anesthetic transperineal (LATP) techniques have made it feasible to perform transperineal biopsy in the office, removing the historical requirement for general anesthesia and a brachytherapy-style stepper and grid [10].

The consequence of this dual evolution is that the comparison most relevant to a contemporary urology service is rarely a clean, single-variable contrast. A unit deciding whether to retool is usually weighing a complete contemporary pathway—transperineal access with MRI-targeted cores, with or without accompanying systematic cores—against the complete historical pathway it would replace, namely transrectal systematic biopsy without MRI targeting. This pragmatic, pathway-level framing is the lens adopted throughout this review. It necessarily blends two variables—route and targeting strategy—but it reflects the real clinical decision, and we draw on the subset of randomized and comparative evidence that isolates each variable wherever possible to avoid conflating them uncritically. It is worth stating at the outset why this distinction matters in practice rather than merely in principle. Several of the most influential early trials varied both factors at once: PRECISION, for example, compared an MRI-targeted pathway against transrectal systematic biopsy, and is rightly cited as transformative evidence for targeting—yet both of its arms used the transrectal route, so it speaks to targeting and not to route [11]. A reader who treats all of the available studies as answering a single question will reach incoherent conclusions; throughout, we signal for each piece of evidence whether the contrast is one of route, of targeting, or of the bundled pathway.

This narrative review is deliberately balanced in emphasis. Rather than advancing a single recommendation, it weights three considerations approximately equally: the detection of clinically significant prostate cancer (defined throughout, unless otherwise stated, as International Society of Urological Pathology [ISUP] grade group ≥ 2), the harms and patient experience associated with each pathway, and the practical and economic realities of implementation. We first describe the evolution of biopsy technique and the imaging substrate that underpins targeting, illustrated with clinical mpMRI examples spanning PI-RADS categories 2 through 5. We then examine, in turn, comparative cancer detection, detection by tumor location, infectious and non-infectious complications, patient-reported and functional outcomes, the choice of fusion technique, cost-effectiveness, special populations, emerging adjuncts to the biopsy pathway, and the divergent positions of major international guidelines [12,13]. We close by identifying the questions that remain genuinely unresolved. This is expressly a narrative rather than a systematic review: it offers a considered synthesis of the principal evidence rather than a protocol-driven, reproducible meta-analysis, and the limitations of that approach are discussed in Section 11.

Throughout, we attribute each piece of evidence to route, to targeting, or to the bundled pathway as the underlying study design allows: trials that hold targeting constant and vary only route (PREVENT, ProBE-PC, PERFECT, TRANSLATE) isolate the effect of route [14,15], whereas those that hold route constant and vary only targeting (PRE-CISION, PRECISE) isolate the effect of targeting [11,16].

Although this is a narrative rather than a systematic review, we followed a structured approach to literature identification. We searched PubMed/MEDLINE, Embase, and the Cochrane Library through May 2026 using combinations of the terms “prostate biopsy,” “transperineal,” “transrectal,” “MRI-targeted,” “fusion biopsy,” “systematic biopsy,” and “PI-RADS.” We prioritized, in order: (i) randomized controlled trials directly comparing transperineal and transrectal access; (ii) systematic reviews and meta-analyses of route or targeting comparisons; and (iii) current guideline statements from the European Association of Urology, the American Urological Association/Society of Urologic Oncology, the European Society for Medical Oncology, and the UK National Institute for Health and Care Excellence. Reference lists of retrieved reviews and included trials were hand-searched for additional relevant studies. Because this is a narrative synthesis rather than a systematic review, no formal risk-of-bias assessment or quantitative pooling was performed, a limitation discussed further in Section 11.

## 2. The Evolution of Prostate Biopsy—Imaging Substrate of Targeting

### 2.1. From Systematic Transrectal Sampling to MRI-Targeted Biopsy

The systematic sextant biopsy introduced in the late 1980s, later extended to ten or twelve cores, established TRUS-guided systematic sampling as the reference standard for prostate cancer diagnosis [5,6]. Its central assumption—that a blind, evenly distributed set of cores would adequately represent the gland—proved too simple once whole-mount prostatectomy series accumulated: tumors clustered in the peripheral zone, but a substantial minority arose anteriorly and in the transition zone, beyond the reach of a laterally directed transrectal needle [7,17]. Extending the template from six to twelve cores improved peripheral-zone yield but could not reach tissue the trajectory never crossed—the conceptual ceiling that both MRI targeting and the transperineal route were separately designed to break. This radiologically and clinically elusive anterior tumor, the “prostatic evasive anterior tumor” [8], became the emblematic failure mode of the blind transrectal approach and the shared motivation for the two innovations described in this and the following section.

The remainder of this subsection concerns only the first of these innovations—MRI targeting—and, because targeting rather than route is not the axis this review is built around, is summarized briefly rather than trial by trial. Landmark validating and randomized studies—including PROMIS, PRECISION, MRI-FIRST, the 4M study, STHLM3-MRI, the NCI fusion series, and PRECISE—established, in designs that varied targeting while holding the transrectal route constant in both arms, that MRI-targeted sampling detects clinically significant cancer at least as reliably as systematic transrectal sampling while substantially reducing the detection of clinically insignificant disease [11,16,18,19,20,21,22,23]; subsequent systematic reviews and meta-analyses have confirmed this pattern across the wider literature [24,25]. In Section 3.1, we summarize these targeting-axis studies and provide trial-level detail. Because every comparison in this group used the transrectal route in both arms, they speak to the value of targeting, not to the choice between transperineal and transrectal access—the comparison this review is further built around.

### 2.2. The Return of the Transperineal Route

Transperineal access is not new; it predates the transrectal route historically but fell out of routine use because early implementations required general anesthesia and specialized equipment such as a brachytherapy-style stepper and grid [10]. Its resurgence has been driven primarily by the infection argument. Because the perineal approach does not breach the rectal mucosa, it largely eliminates the direct inoculation of enteric organisms into the prostate and bloodstream, and a series of studies has shown very low infectious complication rates even when antibiotic prophylaxis is reduced or omitted entirely. The parallel development of freehand local-anesthetic devices has made office-based transperineal biopsy practical, removing the logistical barriers that previously confined the route to the operating theater.

The transperineal route also has a diagnostic rationale. Approaching the gland from the perineum along its cranio–caudal axis provides more direct access to the anterior prostate, the apex, and the transition zone, precisely the regions that transrectal systematic schemes sample least reliably [8,26]. This anatomical advantage underlies much of the location-specific detection data discussed in Section 3.2 and provides a mechanistic explanation for why the route may matter most in men with anteriorly located tumors, including those with prior negative transrectal biopsies [27,28].

McNeal’s zonal model explains why: it partitions the gland into a peripheral zone, where the majority of carcinomas arise and which the transrectal needle samples well; a central zone; and a transition zone, which enlarges with benign hyperplasia and lies anteromedially, beyond the easy reach of a posterolaterally directed needle [17]. Whole-mount radical-prostatectomy series confirm that a substantial minority of tumors originate anteriorly and in the transition zone, and that these anterior-predominant cancers can be of high grade and clinically consequential, so their systematic under-sampling by the transrectal route is not a trivial omission [7,26].

The practical re-engineering of the transperineal route over the past decade is what converted an anatomically attractive but logistically burdensome technique into a routine office procedure. Historical transperineal biopsy used a transrectal ultrasound probe with a brachytherapy-style stepper and a perineal template grid, which mandated lithotomy positioning and, usually, general anesthesia; the template approach also encouraged saturation sampling with large core numbers [10]. The introduction of freehand access devices, which allow the needle to be steered under real-time ultrasound through one or two perineal puncture points without a fixed grid, removed both the grid and the requirement for general anesthesia, allowing the procedure to be performed under local anesthesia in the clinic. This shift matters for the present comparison in two ways. First, it brings the transperineal route onto the same logistical footing as office-based transrectal biopsy, neutralizing what was historically the strongest practical argument for the transrectal approach. Second, by moving away from fixed saturation templates toward targeted and limited systematic sampling, it reduces the core numbers that drive several of the route’s characteristic harms, including procedural discomfort and the periprostatic edema implicated in urinary retention [9].

### 2.3. Multiparametric MRI and PI-RADS: The Imaging Substrate of Targeting

The value of any targeted biopsy pathway is bounded by the quality and interpretation of the MRI that defines the target. Building on European Society of Urogenital Radiology consensus guidelines [29], the Prostate Imaging Reporting and Data System (PI-RADS) provides a standardized framework for acquiring and reporting prostate mpMRI and for scoring each lesion from 1 (clinically significant cancer highly unlikely) to 5 (highly likely). The assessment integrates T2-weighted imaging, diffusion-weighted imaging with its apparent diffusion coefficient (ADC) map, and dynamic contrast-enhanced imaging, and the dominant sequence depends on zonal anatomy: diffusion-weighted imaging governs scoring in the peripheral zone, while T2-weighted morphology governs the transition zone, where benign hyperplastic nodules can otherwise mimic diffusion restriction [30,31,32]. Readers seeking the full sequence-by-sequence scoring logic—*b*-value thresholds, the DCE tie-breaker rule, and so on—are referred to the original PI-RADS v2.1 publication [31].

The clinical importance of the PI-RADS category lies in its strong, broadly monotonic relationship with the probability of harboring clinically significant cancer, with detection rates rising steeply across categories—on the order of 6%, 5%, 19%, 54%, and 84% for categories 1 through 5 in one meta-analysis, with closely concordant figures in others [33,34]. The pooled positive predictive value of a suspicious mpMRI is approximately 40%, with substantial between-study heterogeneity driven by cancer prevalence, scanner quality, and reader experience [35,36,37]—which is precisely why MRI quality is not a peripheral concern, and why the Prostate Imaging Quality (PI-QUAL) score was developed to audit the technical adequacy of the sequences on which the entire targeted pathway depends [38,39].

**Figure 1:**
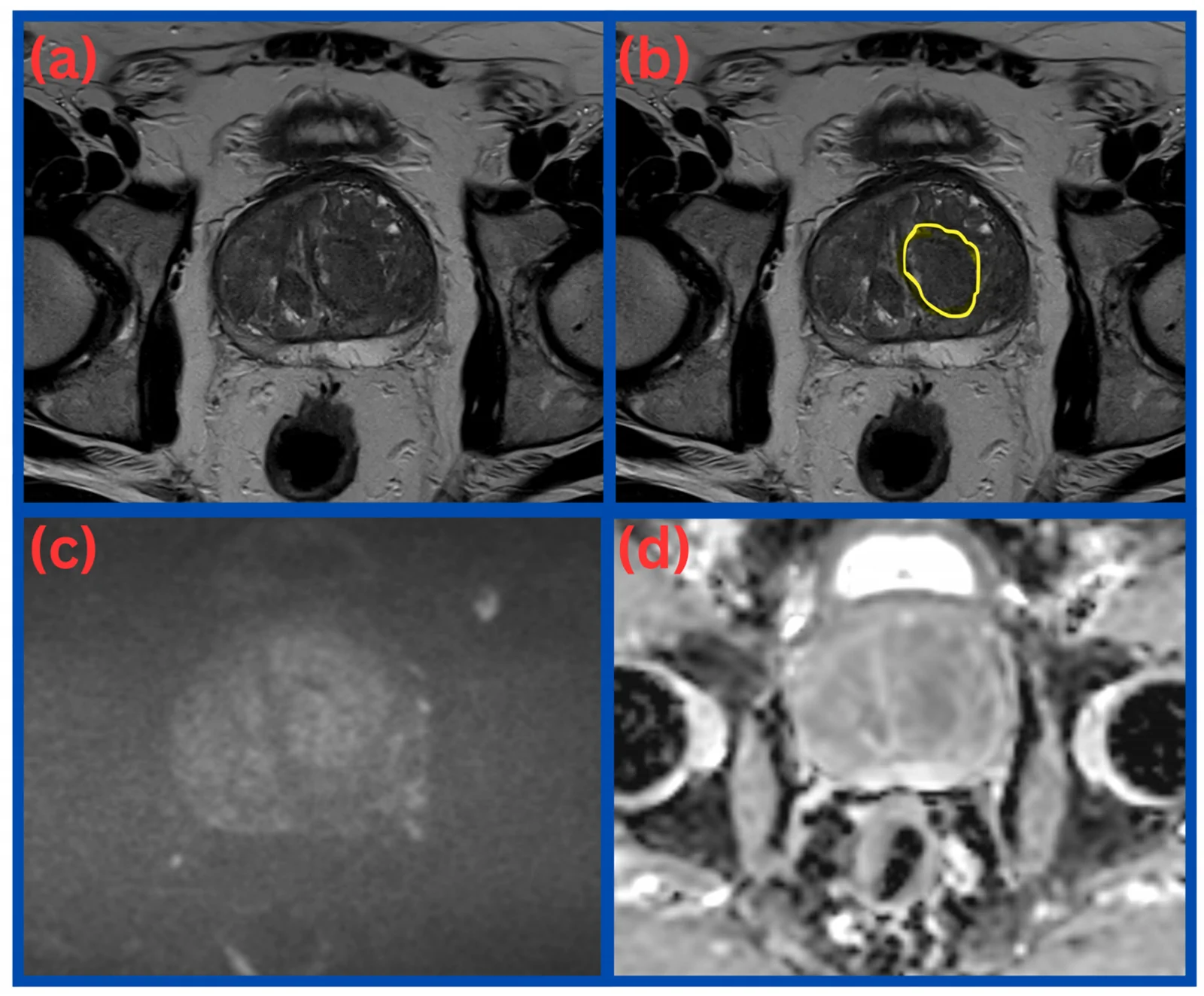
Multiparametric MRI of a PI-RADS 2 appearance in the transition zone. (**a**) Axial T2-weighted image showing multiple well-circumscribed cystic and nodular changes with only mild T2 hypointensity, measuring up to approximately 25 mm and typical of benign nodular hyperplasia, and (**b**) the corresponding axial T2-weighted image with the region of interest outlined in yellow. (**c**) High *b*-value diffusion-weighted image (*b* = 1400 s/mm^2^) and (**d**) the apparent diffusion coefficient (ADC) map demonstrates no convincing diffusion restriction corresponding to the T2 changes. The combination of well-defined margins, encapsulated morphology, and absence of a diffusion correlate is characteristic of a PI-RADS 2 (clinically significant cancer unlikely) assessment.

**Figure 2:**
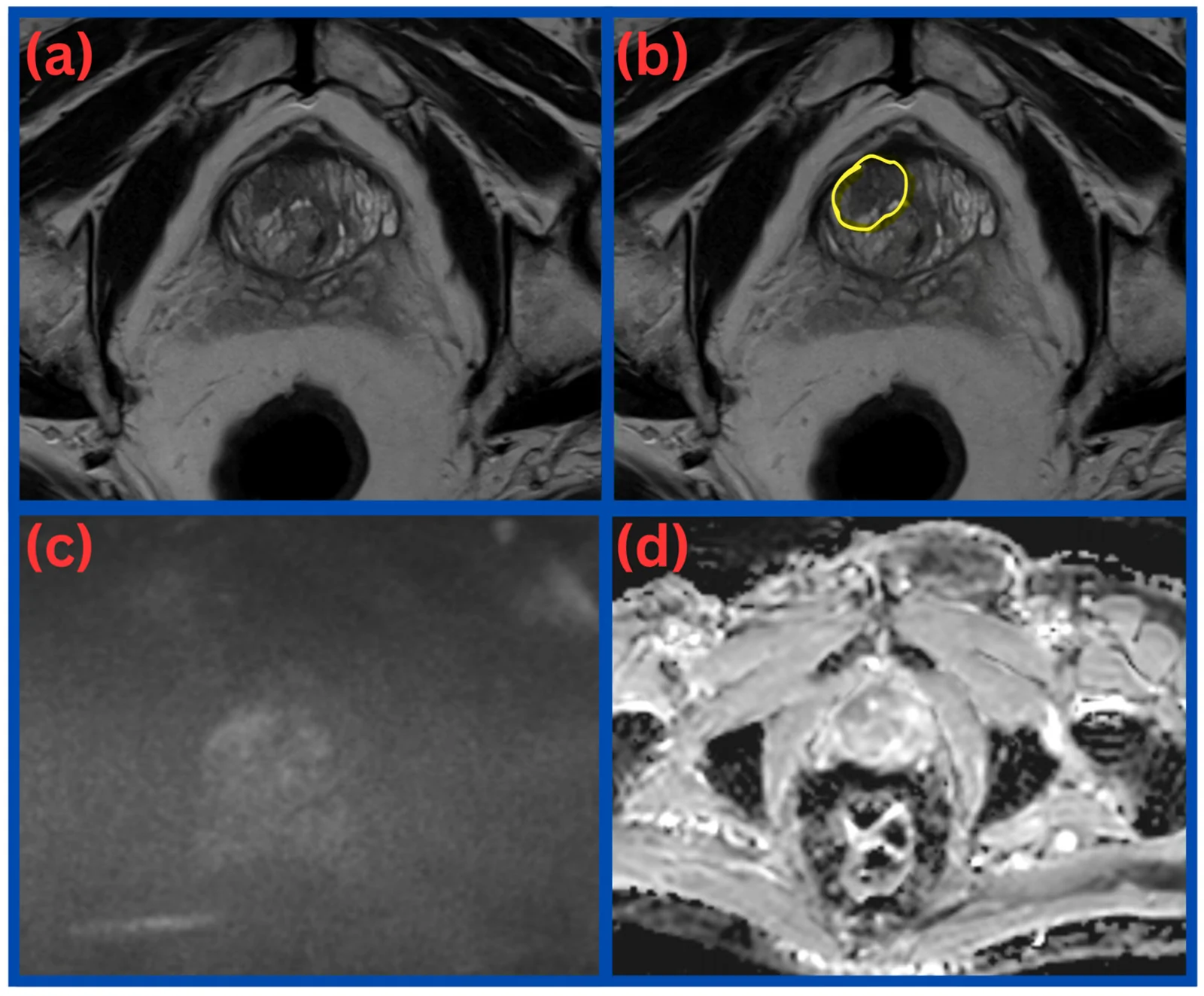
Multiparametric MRI of a PI-RADS 3 lesion. (**a**) Axial T2-weighted image showing a reasonably well-delimited area of T2 hypointensity in the antero-lateral right transition zone, and (**b**) the corresponding axial T2-weighted image with the lesion outlined in yellow. (**c**) High *b*-value diffusion-weighted image (*b* = 1400 s/mm^2^) shows only minimal high signal, and (**d**) the ADC map shows only mild corresponding hypointensity, indicating equivocal (mild) diffusion restriction. The intermediate appearance—T2 hypointensity with only mild diffusion correlate—is characteristic of a PI-RADS 3 (intermediate) assessment, the category for which the decision to biopsy or to use ancillary risk stratification such as PSA density is most finely balanced.

**Figure 3:**
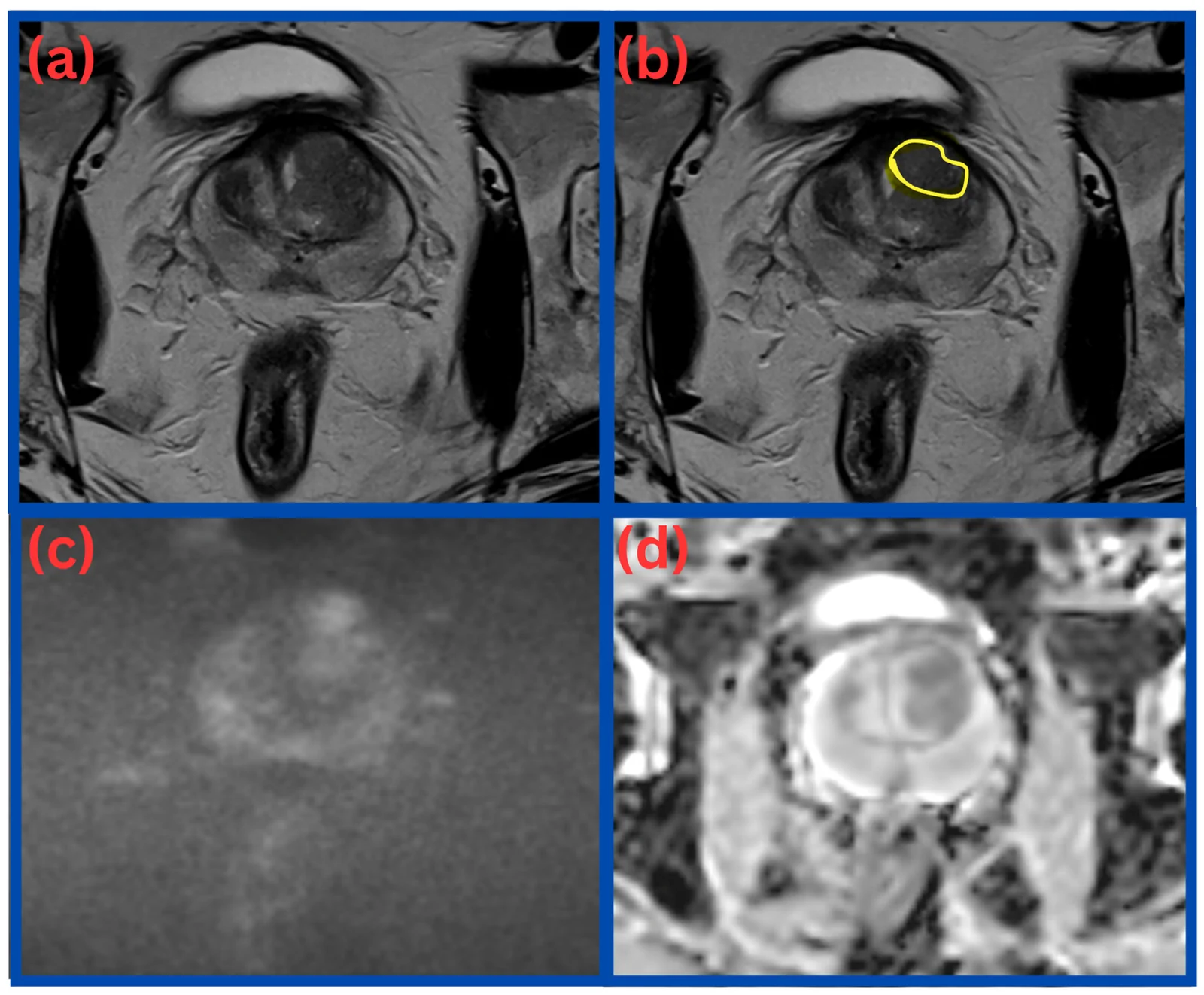
Multiparametric MRI of a PI-RADS 4 lesion. (**a**) Axial T2-weighted image showing a focal area of T2 hypointensity, and (**b**) the corresponding axial T2-weighted image with the lesion outlined in yellow. (**c**) High *b*-value diffusion-weighted image (*b* = 1400 s/mm^2^) demonstrates focal high signal, and (**d**) the ADC map shows corresponding marked hypointensity, together indicating definite diffusion restriction. The combination of a focal, non-circumscribed lesion with clear diffusion restriction corresponds to a PI-RADS 4 (clinically significant cancer likely) assessment, warranting targeted biopsy.

**Figure 4:**
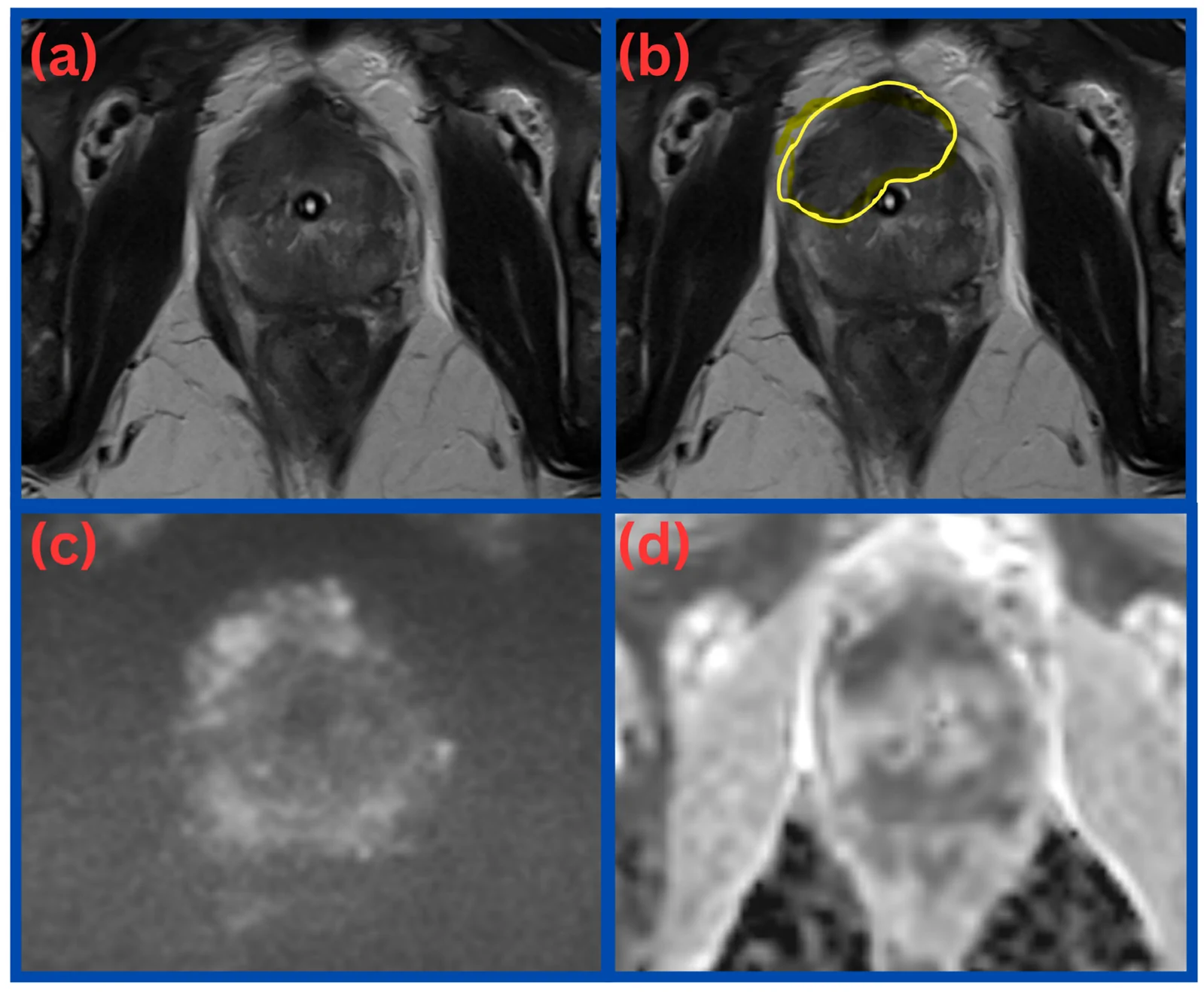
Multiparametric MRI of a PI-RADS 5 lesion with extraprostatic extension. (**a**) Axial T2-weighted image showing a diffusely marginated area of T2 hypointensity in the anterior portion of the transition zone that appears to infiltrate and extend beyond the prostatic capsule, and (**b**) the corresponding axial T2-weighted image with the lesion outlined in yellow. (**c**) High *b*-value diffusion-weighted image (*b* = 1400 s/mm^2^) shows marked high signal, and (**d**) the ADC map shows marked corresponding hypointensity, indicating pronounced diffusion restriction. A lesion of this size and signal characteristics with apparent capsular breach corresponds to a PI-RADS 5 (clinically significant cancer highly likely) assessment. The anterior transition-zone location exemplifies the disease phenotype that transrectal systematic sampling reaches least reliably; Section 3.2 details the supporting quantitative evidence, including a per-patient relative risk of 2.55 (95% CI 1.56–4.16) and an odds ratio of 2.67 (95% CI 1.42–5.00) favoring transperineal detection of anterior, transition-, and central-zone disease, together with a concordant non-significant trend for anterior lesions in the PERFECT randomized trial.

To ground these abstract categories in clinical reality, Figure 1, Figure 2, Figure 3 and Figure 4 present mpMRI examples spanning PI-RADS categories 2 through 5, each drawn from routine clinical practice and displayed across the relevant sequences. They illustrate the progression from a clearly benign appearance, through the genuinely equivocal category 3 lesion, to the unambiguous high-category lesion with extraprostatic extension.

Figure 2 illustrates the diagnostically challenging PI-RADS 3 lesion, the category in which the probability of significant cancer is genuinely intermediate and in which management decisions are most contested. Here, the transition-zone lesion shows hypointense T2 signal with only mild, equivocal diffusion restriction—minimal high signal on high *b*-value DWI and corresponding mild ADC reduction—without the marked changes that would justify upgrading [31].

Figure 3 and Figure 4 demonstrate the high-probability end of the spectrum. In Figure 3, a transition-zone lesion shows the morphological and diffusion features that define PI-RADS 4, with clear T2 hypointensity and unambiguous diffusion restriction—high signal on high *b*-value DWI with corresponding low ADC.

Figure 4 shows a PI-RADS 5 lesion in the anterior transition zone (see the figure legend below for the quantitative rationale). The lesion is large, and shows marked diffusion restriction together with morphological evidence of capsular breach, illustrating both the diagnostic confidence conferred by high-category MRI and the anatomical rationale for an access route capable of reaching anterior disease.

Two points emerge from these examples that are directly relevant to the route-and-targeting question. First, the anterior and transition-zone predilection of several illustrated lesions is not incidental; it reflects a genuine anatomical pattern of disease that interacts with the choice of biopsy route [17,26]. Second, the quality of the underlying sequences—the clarity of the T2 morphology, the conspicuity of the high *b*-value DWI signal, and the reliability of the ADC map—directly governs whether a lesion is correctly categorized and therefore whether it is sampled at all [37,39]. These observations frame the comparative data that follow.

## 3. Comparative Detection of Clinically Significant Prostate Cancer

### 3.1. Overall Detection: Approximate Equivalence with a Modest Transperineal Advantage

When the comparison is framed at the level of overall clinically significant cancer detection, the weight of high-quality evidence points to approximate equivalence between the transperineal and transrectal MRI-targeted approaches, with a small advantage for the transperineal route emerging in the largest trial and in observational cohorts that capture its anterior sampling benefit. A meta-analysis by Uleri and colleagues, pooling studies of MRI-targeted procedures by both routes, found no significant difference in significant-cancer detection (pooled odds ratio [OR] 1.11, 95% confidence interval [CI] 0.98–1.25, *p* = 0.1) [40]. A subsequent meta-analysis restricted to prospective studies, including the principal randomized trials, similarly found no significant difference in significant-cancer detection (OR 0.9, 95% CI 0.7–1.1) and—importantly for the over-diagnosis question—no difference in the detection of ISUP grade group 1 (insignificant) cancer (OR 1.1, 95% CI 0.8–1.4) [41].

The randomized evidence comparing the routes directly is now substantial, and it is instructive precisely because the individual trials do not all point the same way. The PREVENT trial randomized men to transperineal biopsy without antibiotic prophylaxis or to transrectal biopsy with targeted prophylaxis and found similar significant-cancer detection (53% versus 50%; adjusted difference 2%, 95% CI −6 to 10) alongside a lower infection rate in the transperineal arm that did not reach statistical significance in the primary analysis (0% vs. 1.4%; *p* = 0.059) [14]. The single-center ProBE-PC trial, which used the same fusion platform for both arms, found no significant difference in grade group ≥ 2 detection (43.2% transperineal versus 47.1% transrectal) and, notably, no sepsis in either arm [42,43]. The PERFECT trial, by contrast, was a non-inferiority study whose primary endpoint was confined to MRI-targeted cores in MRI-positive men, and it failed to demonstrate non-inferiority of the transperineal route for targeted significant-cancer detection (47.2% versus 54.2%; absolute difference −7.0% against a prespecified 5% noninferiority margin, so noninferiority was not demonstrated)—a result driven, as discussed below, by superior transrectal sampling of posterior lesions [44].

The largest randomized comparison, the multicenter TRANSLATE trial, random-ized 1126 biopsy-naive men with pre-biopsy MRI to local-anesthetic transperineal or transrectal biopsy and reported the opposite signal to PERFECT: grade group ≥ 2 cancer was detected in 60% of the transperineal arm versus 54% of the transrectal arm (OR 1.32, 95% CI 1.03–1.70, *p* = 0.031), a statistically significant advantage for the trans-perineal route [15]. The apparent discrepancy between PERFECT and TRANSLATE is largely explained by design: PERFECT confined its primary endpoint to targeted cores in MRI-positive men, whereas TRANSLATE compared complete pathways including systematic sampling, so the trials are answering subtly different questions [15,44]. TRANSLATE itself offers a quantitative sense of how much of this pathway-level advantage reflects the additive value of systematic sampling rather than route-specific targeting. Across the trial’s full modified intention-to-treat population (both arms combined), MRI-targeted cores alone detected GGG ≥ 2 cancer in 472 of 1126 participants (41.9%), while a further 151 participants (13.4%) had their significant cancer detected only by systematic cores—that is, roughly one in four of all clinically significant cancers found in the trial would have been missed had systematic sampling been omitted [15]. This confirms that the additive contribution of systematic sampling is substantial in absolute terms; however, TRANSLATE’s published report does not decompose this figure by randomized arm, so it establishes the general magnitude of the systematic-sampling effect without yet fully separating, within the transperineal arm specifically, how much of its advantage reflects route-specific anterior access versus the general value of combining targeted with systematic cores. The trial investigators note that further analysis of detection by targeted, peri-lesional, and systematic cores is planned [15]. Taken together, the randomized and propensity-matched evidence supports overall equivalence, with a modest transperineal advantage emerging in the largest pathway-level comparison and in observational datasets that capture the route’s anterior sampling benefit.

Observational meta-analyses that include large non-randomized cohorts tend to detect this modest transperineal advantage more readily. Wu and colleagues, pooling fifteen studies and 8826 patients, reported higher overall cancer detection with the transperineal route (relative risk [RR] 1.25, 95% CI 1.12–1.39) and higher per-patient significant-cancer detection (RR 1.37, 95% CI 1.16–1.61) [45]. A propensity-score-matched analysis across fifteen European centers by Diamand and colleagues similarly found greater significant-cancer detection with the transperineal route (grade group ≥ 2: 51% versus 45%, OR 1.37, 95% CI 1.15–1.63; grade group ≥ 3: 29% versus 23%, OR 1.38, 95% CI 1.13–1.67) [46]. The most parsimonious reconciliation of the randomized and observational studies is that the transperineal advantage is real but spatially concentrated—it accrues chiefly in the anterior and apical gland—so its visibility in any given dataset depends on how many such tumors that cohort contains and on how intensively each arm samples. Table 1 summarizes the principal comparative studies.

Two methodological features of this literature shape how the pooled estimates should be read. The first is the era effect: studies conducted before the widespread adoption of pre-biopsy MRI compared systematic sampling in both arms, whereas more recent studies incorporate MRI as a triage and targeting tool, so pooling across eras blends fundamentally different pathways and inflates heterogeneity [24]. The second is the confounding between route and sampling intensity already noted: transperineal protocols, particularly template-based ones, often take more cores than transrectal systematic schemes, so an apparent detection advantage may partly reflect more extensive sampling rather than the route itself. The propensity-matched and randomized analyses that attempt to control for these factors tend to report smaller, often non-significant, differences than the unadjusted observational pools [40,41]—a pattern entirely consistent with a true effect that is modest, spatially concentrated, and partly mediated by core number. The over-diagnosis question deserves separate emphasis, because the detection of insignificant cancer carries the downstream harms of unnecessary surveillance or treatment. Here, the evidence is reassuring and route-agnostic: prospective-study meta-analysis finds no significant difference between routes in the detection of grade group 1 disease [41]. This contrasts instructively with the targeting question, where the central benefit of MRI demonstrated in PRECISION was precisely a reduction in insignificant-cancer detection relative to systematic sampling [11], a further illustration of why route and targeting must be reasoned about separately even when they are adopted together.

### 3.2. Detection by Tumor Location: Where the Route Matters Most

The clearest and most reproducible difference between the routes is not in aggregate detection but in the spatial pattern of what each route finds. The transperineal trajectory samples the anterior gland, the apex, and the transition zone more effectively, while the transrectal trajectory, approaching the gland posteriorly, can sample posterior peripheral-zone lesions at least as well and in some analyses better.

The PERFECT trial provides the most direct randomized evidence for the posterior advantage of the transrectal route: on per-lesion analysis, posterior lesions were detected significantly more often transrectally (59.0% versus 44.3%, *p* = 0.044), while anterior lesions trended toward better transperineal detection without reaching significance in that trial [44]. Conversely, the multi-institutional meta-analysis by Zattoni and colleagues found the transperineal route significantly more likely to detect significant cancer in the apex, the transition or central zone (OR 2.67, 95% CI 1.42–5.00), and the anterior zone, with the largest adjusted effects reported for the anterior and apical locations [47]. Wu and colleagues quantified the anterior advantage meta-analytically, with a per-patient relative risk of 2.55 (95% CI 1.56–4.16) favoring transperineal detection of anterior significant cancer [45].

This spatial complementarity has a clear anatomical basis. As Section 2.2 establishes, the posterolateral needle trajectory of the transrectal approach reaches the anterior gland poorly; whole-mount and prostatectomy series further confirm that anterior-predominant tumors constitute a distinct and clinically consequential subset [26]. Approximately one-fifth to one-quarter of prostate cancers arise in the transition zone, so this is not a negligible subgroup [17]. The lesion illustrated in Figure 4, an anterior transition-zone PI-RADS 5 cancer with capsular breach, is exactly the kind of disease for which route selection is most consequential: a posteriorly directed systematic scheme may miss or under-grade it, whereas a perineal approach reaches it directly. For lesions confined to the posterior peripheral zone, by contrast, the transrectal route remains entirely adequate and may offer marginally more reliable sampling [44].

## 4. Harms and Safety

### 4.1. Infectious Complications: The Central Argument for the Transperineal Route

If detection is broadly a draw, infection is where the two routes diverge most sharply, and it is the single consideration that has done most to drive the international migration toward transperineal biopsy. The microbiological substrate makes the mechanism explicit. The rectum harbors a dense population of Gram-negative enteric organisms, predominantly Escherichia coli, and the transrectal needle inevitably carries some of this flora into the prostate and, through the rich periprostatic venous plexus, into the bloodstream. For decades this was contained by fluoroquinolone prophylaxis, but the progressive emergence of fluoroquinolone-resistant rectal flora—driven by cumulative antibiotic exposure across the population—eroded that containment, so that the organisms most likely to be inoculated became precisely those least likely to be covered [9,48].

Historical and registry data document the scale of the problem. The multinational Global Prevalence Study of Infections in Urology recorded febrile urinary infections and hospitalization in a few percent of men despite near-universal fluoroquinolone prophylaxis [49], and single-system series documented a marked rise in post-biopsy infective complications over the 2000s as resistance increased [50]. The regulatory restriction of systemic fluoroquinolones in Europe from 2019, issued because of disabling and potentially irreversible adverse effects, further constrained the standard transrectal prophylaxis regimen and accelerated interest in an approach that could sidestep the issue altogether [51]. Within the transrectal route, mitigation strategies—povidone-iodine rectal preparation, augmented or rectal-swab culture-directed prophylaxis—reduce but do not eliminate the infectious risk, and they add cost and complexity [4,52,53].

The transperineal route addresses the problem at its source, and comparative meta-analyses show that transperineal biopsy is associated with significantly lower rates of infection than the transrectal route [54]. That pooled estimate, however, needs to be read against a specific source of heterogeneity: the transrectal comparator arms feeding these pooled analyses span a wide range of prophylaxis intensity, from single-agent empirical fluoroquinolone prophylaxis to culture-directed, augmented combination regimens, and the infection rate actually achievable on the transrectal side depends heavily on which of these is in use [52]. This is not a minor caveat—in the most rigorously conducted recent head-to-head trials, which used optimized transrectal prophylaxis, the absolute infection gap narrows substantially. Both ProBE-PC and PREVENT recorded very low infection rates in their transrectal arms: ProBE-PC reported no sepsis in either arm [43], and in PREVENT the transperineal-without-antibiotics arm was only numerically, not statistically, lower in infections than its optimally prophylaxed transrectal comparator (0% vs. 1.4%; *p* = 0.059) [14]. Read together, these findings suggest that the literature-wide infection advantage for the transperineal route is real and consistent in direction, but that its magnitude is best understood as an average across a heterogeneous set of transrectal prophylaxis practices rather than a fixed, prophylaxis-independent property of the route itself; at a center that already practices optimized, ideally culture-directed, transrectal prophylaxis, the achievable gap may be materially smaller than pooled estimates from mixed-quality-prophylaxis literature would suggest [52].

Independent of comparator-arm prophylaxis, the transperineal route’s own safety with reduced or no prophylaxis is well established: studies of transperineal biopsy with and without antibiotic prophylaxis show very low infection rates either way [55,56], and the NORAPP randomized trial found that omitting prophylaxis for transperineal biopsy was non-inferior to giving it, with no sepsis or hospitalized urinary infection in either arm and a number-needed-to-treat of 137 to prevent a single non-hospitalized infection [57].

This licenses a broader, population-level argument that holds independently of the per-patient comparison at any single well-resourced center: because the transperineal route permits prophylaxis to be reduced or omitted without an increase in serious infection, it reduces aggregate antibiotic consumption and the selection pressure for resistance across a population [57]—part of why the EAU’s preference for the route rests on antimicrobial stewardship as much as on the per-patient infection rate [12].

### 4.2. Non-Infectious Complications

Beyond infection, the complication profiles of the two routes differ in predictable, mechanistically sensible ways. Rectal bleeding is far less common with the transperineal route, which does not traverse the rectum; comprehensive systematic reviews of biopsy complications place the odds of rectal bleeding substantially lower for transperineal biopsy [9,58]. Hematuria and hematospermia rates are broadly similar between routes, although the single-center ProBE-PC trial recorded somewhat more minor hematuria after transperineal biopsy and more rectal bleeding after transrectal biopsy, neither requiring intervention [43].

Acute urinary retention is the one non-infectious outcome on which the literature is genuinely inconsistent, and it deserves to be reported as such rather than smoothed over. Some analyses find lower retention with the transperineal route, the health-technology assessment underpinning current UK guidance reports that local-anesthetic transperineal biopsy carries a higher burden of urinary-retention-type symptoms, and randomized-only comparisons find no significant difference [14,59]. The most plausible explanation for this divergence is heterogeneity in template design and core number: saturation transperineal templates with many cores plausibly increase periprostatic edema and retention risk, whereas targeted or limited core transperineal schemes do not. The signal, in other words, is probably an artifact of sampling intensity rather than of the route itself [9]. Table 2 summarizes the comparative harm profile.

## 5. Patient Experience and Functional Outcomes

A balanced appraisal must weigh how the procedure feels and what it leaves behind, not only what it finds. Here the ledger tilts modestly toward the transrectal route. The transperineal approach is more frequently experienced as painful: in the TRANSLATE trial, 38% of men reported moderate or severe immediate pain with local-anesthetic transperineal biopsy versus 27% with transrectal biopsy (OR 1.84, 95% CI 1.40–2.43), although overall acceptability remained high in both arms [15]. Importantly, transperineal pain is modifiable by anesthetic technique: the APROPOS randomized trial demonstrated that a perineal nerve block provides superior pain control to a periprostatic block for men undergoing transperineal biopsy, and pain increases with the number of cores taken [60]. This means the discomfort cost of the transperineal route is not fixed, and can be reduced through deliberate attention to the anesthetic approach and judicious limitation of core number.

Functional outcomes, by contrast, appear largely independent of route and are generally transient. Erectile function declines modestly after prostate biopsy by either route and recovers over the following months: a meta-analysis found a mean reduction of roughly two points on the abbreviated International Index of Erectile Function at one month, with recovery by three months [61]. TRANSLATE found similar urinary and sexual function at four months between the two arms, reinforcing the view that any functional difference between the routes is short-lived and clinically minor [15]. For the patient, then, the experiential trade-off is reasonably clear, and is best framed as material for shared decision-making rather than as a verdict: the transperineal route trades a somewhat more uncomfortable procedure for a substantially lower risk of infection and rectal bleeding. A man who is highly anxious about infection, who has had a prior infectious complication, or who lives far from acute care may reasonably prioritize the transperineal route’s infection advantage; a man particularly apprehensive about perineal discomfort, or who tolerated a previous transrectal procedure well, may weigh the calculus differently, especially where local transrectal infection rates are low. The clinician’s role is to make these trade-offs explicit and quantified rather than to present either route as categorically superior.

The patient-experience literature also bears on adherence to surveillance and re-biopsy, which is easy to overlook when focusing on a single procedure. Prostate cancer diagnosis is increasingly iterative: men on active surveillance undergo repeated biopsies over years, and men with a negative initial biopsy but persistent suspicion may be advised to undergo another. A route that is better tolerated, or that is perceived as less threatening, may improve willingness to return for necessary repeat sampling, while one that is remembered as painful may deter it. The TRANSLATE finding that acceptability remained high in both arms despite greater immediate discomfort with the transperineal route is therefore reassuring [15], as is the demonstration that procedural pain can be materially reduced by appropriate regional anesthesia rather than being an immutable feature of the perineal approach [60]. The transient and recoverable nature of the functional effects on urinary and erectile function further supports the view that, for most men, the experiential differences between the routes—while real—are unlikely to dominate a well-informed decision that also weighs infection risk and diagnostic yield [61].

## 6. The Choice of Targeting Technique

Having weighed the comparative experience and functional cost of each route, we turn next to a related but analytically separate question: once mpMRI has identified a target, which technique should be used to sample it? Targeting can be achieved by cognitive registration, in which the operator mentally maps the MRI lesion onto the real-time ultrasound image; by software-assisted fusion, in which dedicated platforms co-register the two image sets; or by in-bore biopsy performed within the MRI scanner. A recurring question is whether the choice among these materially affects yield, and the evidence suggests that it does not, at least not consistently. A systematic review comparing in-bore, software-fusion, and cognitive-registration techniques found no clearly superior method for clinically significant cancer detection [62]. The FUTURE trial randomized men with prior negative biopsies among cognitive, software-fusion, and in-bore targeting and found no significant difference in significant-cancer detection across the three arms [63]. The SmartTarget within-person trial found that visual registration and software fusion were complementary rather than competitive, with the combination of both achieving the highest detection—an argument for redundancy rather than for the superiority of any one method [64], and the PROFUS trial similarly found fusion and visual-estimation targeting to perform comparably for significant disease [65].

Meta-analytic comparison of cognitive versus software fusion shows at most a non-significant trend favoring software fusion, with considerable inter-study variability [66]. In-bore biopsy may yield slightly fewer insignificant cancers in some series but is resource-intensive and slow, and it is best reserved for the minority of cases in which previous targeted attempts have failed or in which the lesion is small, anterior, and difficult to reach. The practical implication is that the fusion modality does not interact strongly with the route: a unit can reasonably select the targeting method according to local expertise and equipment, and should invest its attention instead in MRI quality and PI-RADS interpretation, which exert a larger influence on yield than the targeting platform [37,39].

A practical corollary is that a unit need not acquire expensive software-fusion hardware to deliver a high-quality targeted pathway: the principal advantages of software fusion lie in documentation, auditability of needle placement, and the training of less experienced operators rather than in a decisive detection benefit. The sensible posture for most services is to choose the modality that fits their case mix and expertise, and to recognize that the marginal gains from upgrading the fusion platform are smaller than those from improving MRI acquisition and reporting [37].

## 7. Cost-Effectiveness and Implementation

Whichever targeting technique is chosen, adopting the transperineal route at all is, for many units, first an economic and logistical decision, and it is to that question that we turn next. The economic case for the transperineal route rests heavily on health-technology assessment conducted within the United Kingdom (UK)’s National Health Service (NHS), and its conclusions are best understood as context-dependent rather than universally applicable: reimbursement structures, baseline antibiotic costs, infection-related admission costs, and procedural overhead all vary substantially across health systems, and each materially affects where the cost-effectiveness threshold falls. Within that UK context, however, the economic case has increasingly favored the transperineal route, driven less by the procedure itself than by the downstream costs it avoids—chiefly infection-related admissions and antibiotic use—and by the diagnostic yield that reduces the need for repeat sampling. The UK’s National Institute for Health and Care Excellence, drawing on a dedicated health-technology assessment, concluded that local-anesthetic transperineal biopsy using a freehand device meets conventional cost-effectiveness thresholds relative to transrectal biopsy, with incremental cost-effectiveness ratios well below the customary willingness-to-pay threshold across risk subgroups, and was most cost-effective in higher-risk, MRI-selected men in whom diagnostic yield is greatest [59]. These conclusions were derived within a UK NHS costing framework and a UK-specific antibiotic-resistance and hospital-admission-cost environment, and should not be assumed to transfer unchanged to health systems with different reimbursement models, antibiotic pricing, or infection-related costs of care. A device-level economic evaluation reached a compatible conclusion, finding that transperineal biopsy is cost-effective below modest per-procedure device costs, with the saving driven by reduced infection-treatment costs and the quality-of-life gains from fewer infections [67].

Implementation considerations reinforce the economic ones. Freehand local-anesthetic transperineal biopsy can be performed in the office without general anesthesia, reducing theater time and waiting lists relative to historical general-anesthetic transperineal techniques, while the avoidance of fluoroquinolone prophylaxis aligns with antimicrobial stewardship priorities [51]. A learning curve exists—procedural metrics such as operative time improve over roughly the first several dozen to ninety cases—but significant-cancer detection appears stable across the learning curve, suggesting that diagnostic quality is reached earlier than procedural efficiency [68]. Services contemplating a transition should therefore plan for a period of mentored proctoring and should not judge the technique by its earliest cases; the reward is a procedure that can be delivered in the office, frees operating-theater capacity, and reduces reliance on antibiotics whose effectiveness is being eroded by resistance.

The economic argument interacts with the diagnostic-yield argument in a way that is easy to overlook. Because the value of a biopsy pathway depends not only on the cost of the index procedure but on the cost of everything that follows from getting the diagnosis wrong, a pathway that detects significant cancer more reliably and over-detects indolent disease less often generates downstream savings that dwarf the marginal cost of the procedure itself. A missed anterior tumor may surface later as a more advanced and more expensive cancer; an over-detected indolent tumor may commit a man to years of surveillance or to treatment he did not need. The health-technology assessment underpinning current UK guidance captured this by modeling the transperineal route’s cost-effectiveness as greatest in precisely the higher-risk, MRI-selected men in whom diagnostic yield matters most, and weakest in low-prevalence groups where the marginal diagnostic benefit is small [59]. The clinical implication is that the economic case for adopting the transperineal route is strongest for services handling a high proportion of MRI-positive, higher-risk referrals, and more marginal for services dominated by low-suspicion cases in which the choice of route has less leverage on outcomes.

## 8. Special Populations

The route-and-targeting decision is not uniform across patients; several subgroups shift the balance. Men with a prior negative transrectal biopsy and persistent suspicion are an instructive case, because the tumors missed by the initial biopsy are disproportionately anterior and apical—precisely the disease the transperineal route reaches best. Transperineal template and targeted approaches detect a meaningful fraction of cancers missed by prior transrectal sampling, and the cancers so detected are disproportionately anterior and apical [27,28]. In this setting, the addition of MRI targeting addresses the localization problem while the transperineal route addresses the access problem, so the two innovations are complementary rather than redundant.

Men with large prostates and anteriorly located lesions similarly benefit from the transperineal trajectory, for the reasons already established in Section 2.2 [26]. The active-surveillance population raises a related consideration: here the clinical question is not whether cancer is present but whether it has been correctly graded, and reliable sampling of the anterior gland guards against the under-grading that would falsely reassure both patient and clinician. Finally, there is a biologically grounded hypothesis that men of African ancestry, who experience higher prostate cancer mortality and adverse oncologic outcomes, could derive disproportionate benefit from an access route that samples the anterior gland reliably [69]. This hypothesis is plausible but not yet established by prospective route comparison data, and it is currently insufficient to support route selection on the basis of ancestry alone. It is, however, a reminder that the generalizability of the principal randomized trials is constrained: TRANSLATE, for example, enrolled a predominantly White British population, so its detection advantage may not transfer unchanged to more diverse settings [15].

The active-surveillance setting merits closer attention because it inverts the usual diagnostic question and thereby changes the relative value of the two routes. In a man being monitored for known low-risk disease, the clinical task is not to establish whether cancer is present but to detect, as early and reliably as possible, any component of higher-grade disease that would prompt a change in management. Under-sampling of the anterior gland is especially treacherous in this context, because a falsely reassuring biopsy can delay treatment of a significant anterior tumor that was present but unsampled. The transperineal route’s superior access to anterior and apical tissue is therefore of particular value during surveillance, where the cost of a missed anterior lesion is borne over time [27,28]. The same reasoning applies, with even greater force, to men who harbor a high pre-test probability of anterior disease on anatomical or demographic grounds, including those with large transition zones and the anterior-predominant tumors documented in prostatectomy series [26]. These considerations do not amount to an absolute indication for the transperineal route in surveillance, but they tilt the individualized calculus toward it whenever anterior disease is a material concern.

It is useful, finally, to situate the route-and-targeting decision within the wider diagnostic workflow, because the biopsy is one step in a sequence whose earlier stages constrain it. The modern pathway begins not with the needle but with risk stratification—age, family history, PSA and its derivatives, and increasingly biomarkers and risk models—which determines who proceeds to MRI [70,71]. The MRI then performs a triage function, identifying the men and the lesions that warrant sampling and, through the PI-RADS category and ancillary measures such as PSA density, informing whether biopsy can be safely deferred [35]. Only at this point does the question of route and targeting arise, and by then much of the diagnostic work has already been done by the imaging. This sequencing has two implications. First, the marginal value of any biopsy approach depends on the quality of the steps that precede it; a meticulous transperineal biopsy of a target that should never have been biopsied, or that was mischaracterized on a poor-quality scan, cannot rescue a flawed pathway [37]. Second, the harms of biopsy are incurred only by those who proceed to it, so anything that safely reduces the number of unnecessary biopsies—high-quality MRI with judicious use of PI-RADS and PSA density, supported where available by biomarkers—reduces aggregate harm regardless of which route is then chosen. The route decision is important but bounded: it determines how well and how safely a justified biopsy is performed, not whether the biopsy was justified in the first place.

## 9. Emerging Adjuncts to the Biopsy Pathway

The route-and-targeting question sits within a diagnostic pathway that is itself evolving, and several adjuncts are reshaping the context in which biopsy is performed. Upstream of biopsy, blood- and urine-based biomarkers refine the decision of whom to biopsy at all. The Prostate Health Index and the four-kallikrein (4Kscore) panel both improve the specificity of significant cancer prediction over PSA alone [70,72], while the Stockholm3 model, combining clinical, protein, and genetic markers, has been shown in population screening to reduce unnecessary biopsies when used ahead of or alongside MRI [71]. These tools do not bear directly on the choice of route, but they shrink and enrich the population that proceeds to biopsy, thereby raising the pre-test probability of disease and improving the yield of whichever biopsy approach is then used.

Precision diagnosis is also becoming multi-modal in a second sense: combining imaging with molecular and genomic characterization rather than imaging alone. At the pre-biopsy stage, combining biparametric MRI findings with PSA-derived indices such as the free/total PSA ratio and PSA density has been shown to improve prediction of both any prostate cancer and clinically significant disease specifically, reinforcing that imaging and simple serum biomarkers are complementary rather than competing inputs to the decision to biopsy [73]. Beyond detection, multi-omics approaches are beginning to characterize the molecular drivers of prostate cancer behavior: systematic analysis of mutation, methylation, and expression profiles has identified a panel of candidate aneuploidy driver genes associated with prostate cancer progression, metastasis, and the tumor immune microenvironment [74], and integrated multi-omics profiling combining bulk and single-cell transcriptomic data has been used to build immune-centric prognostic models that stratify patients by risk and by subtype-specific patterns of drug resistance [75]. These molecular layers do not currently bear on the choice of biopsy route or targeting technique, but they connect to this review in a structural sense: every such analysis depends on obtaining adequate, representative tumor tissue, which is exactly what the route and targeting decisions discussed throughout this review are designed to secure. As precision prognostic and drug-resistance models mature, the anatomical representativeness of the biopsy sample—not only the binary question of cancer detection—may become an increasingly important criterion by which biopsy pathways are judged.

At the targeting step itself, two technologies are likely to influence future practice. High-resolution micro-ultrasound offers real-time imaging at far greater resolution than conventional ultrasound and has been evaluated as an alternative or adjunct to MRI fusion; the OPTIMUM randomized trial found micro-ultrasound-guided biopsy non-inferior to MRI-guided biopsy for the detection of grade group ≥ 2 cancer, raising the prospect of a streamlined, single-visit targeted pathway that does not depend on a separately acquired MRI [76]. In parallel, artificial intelligence systems for prostate MRI interpretation are maturing rapidly; the international PI-CAI study found that a state-of-the-art AI system was non-inferior to radiologists for significant-cancer detection on MRI, suggesting a future role in standardizing interpretation and mitigating the reader-dependence that currently contributes to the variable positive predictive value of PI-RADS [36,77]. Both technologies are relevant to the present comparison chiefly because they act on the targeting and case-selection steps rather than on the route, and a high-quality target—however generated—remains the precondition for a successful biopsy by either route.

## 10. Divergent Guideline Recommendations

The unsettled state of the evidence is mirrored in the divergence among major guidelines, which have weighted the same trials differently. The European Association of Urology has moved furthest, issuing a strong recommendation to perform prostate biopsy by the transperineal route on the grounds of lower infectious risk and better antibiotic stewardship, alongside strong recommendations for pre-biopsy MRI and against fluoroquinolone prophylaxis [12]. The European Society for Medical Oncology likewise positions the transperineal route favorably within its diagnostic recommendations [78]. In the UK, the National Institute for Health and Care Excellence recommends local-anesthetic transperineal biopsy using freehand devices as an option, supported by its cost-effectiveness analysis, while its broader diagnostic guidance establishes pre-biopsy mpMRI as the standard first investigation [59,79].

The American Urological Association, jointly with the Society of Urologic Oncology, has taken a more permissive position, stating that clinicians may use either the transrectal or the transperineal route—a conditional recommendation reflecting the view that the randomized detection and, in optimally prophylaxed settings, infection data do not yet compel a single answer [13,80]. This transatlantic divergence is not a failure of evidence synthesis so much as a faithful reflection of it: where one body weights the population-level infection and stewardship benefits most heavily, it arrives at a strong transperineal preference, and where another weights the per-patient equivalence in well-resourced trial settings, it preserves clinician discretion. For the individual practitioner, the divergence underscores that the decision remains, appropriately, one of clinical judgment informed by local infection rates, available expertise, lesion location, and patient preference.

## 11. Synthesis and Limitations of the Evidence

Stepping back from the individual outcomes, a coherent picture emerges. At the level of overall significant cancer detection, the contemporary transperineal MRI-targeted pathway and its transrectal comparator are close to equivalent, with the largest pathway-level randomized trial and the observational literature favoring the transperineal route by a modest margin concentrated in the anterior and apical gland [15,40,46]. At the level of harms, the transperineal route offers a clear and consistent reduction in infection and rectal bleeding, purchased at the cost of somewhat greater procedural discomfort that is itself modifiable by anesthetic technique [54,60]. At the level of implementation, the transperineal route is office-feasible, cost-effective, and aligned with antimicrobial stewardship, with a learning curve that affects efficiency more than diagnostic quality [59,68]. The choice of fusion technique is secondary to MRI quality and interpretation [62].

A fuller account of the limitations of the evidence base is owed to the reader. The comparative literature is heterogeneous along almost every axis that matters. Definitions of clinical significance vary; although ISUP grade group ≥ 2 has become the dominant threshold, older studies used alternative criteria that are not interchangeable. Core number and template design vary widely, and because both detection and several harms scale with the number of cores, comparisons that do not hold core number constant confound the effect of route with the effect of sampling intensity. Anesthetic approach varies, with older transperineal series performed under general anesthesia and modern ones under local anesthesia. Prophylaxis regimens in the transrectal comparator arms range from empirical fluoroquinolones to culture-directed augmented combinations, which materially affects the infection contrast [52]. And operator experience varies, with single-center trials such as ProBE-PC reflecting a particular institution’s performance rather than mature multicenter practice [43].

These sources of heterogeneity are not merely technical caveats; they actively explain the apparent contradictions in the literature. The divergence between PERFECT, which found the transperineal route inferior for targeted detection of predominantly posterior lesions, and TRANSLATE, which found the local-anesthetic transperineal pathway superior overall, dissolves substantially once one notes that the trials compared different things—MRI-targeted cores as the primary endpoint in MRI-positive men versus complete pathways including systematic sampling [15,44]. Similarly, the inconsistent direction of the urinary-retention signal across analyses is most parsimoniously read as an artifact of varying core number and template design rather than a stable property of either route. The appropriate posture is therefore not to seek a single decisive effect estimate but to weigh a pattern of evidence: broad detection equivalence with a modest, spatially concentrated transperineal advantage; a consistent and mechanistically coherent transperineal benefit for infection and rectal bleeding; and a modest, modifiable transperineal cost in procedural discomfort. It must also be acknowledged that this is a narrative rather than a systematic review: it does not rest on a protocol-driven exhaustive search, formal risk-of-bias assessment, or quantitative pooling, and readers seeking pooled effect sizes should consult the cited meta-analyses directly, bearing in mind the era effects and the confounding between route and targeting that complicate their interpretation [24,41].

A consideration that cuts across every comparison in this review is that the entire targeted pathway, by whichever route, is only as good as the MRI on which it rests, and MRI interpretation is neither perfectly reproducible nor uniformly available. The pooled positive predictive value of a suspicious mpMRI for significant cancer is on the order of 40%, and it varies widely across centers and readers [35,36]. The PI-RADS 3 lesion exemplified in Figure 2 is the sharpest expression of this uncertainty: it is, by definition, the category in which the probability of significant cancer is intermediate and in which inter-reader disagreement is greatest, so that the decision to biopsy, to defer with PSA-density stratification, or to repeat imaging is genuinely finely balanced. The transition zone, where benign hyperplasia generates a complex baseline signal, is especially demanding to read, and it is also—uncomfortably—the zone in which the route-selection question matters most, since anterior transition-zone tumors are exactly those the transrectal approach reaches least well [8,31]. This is why image quality is a precondition for the whole enterprise rather than a technical afterthought: the PI-QUAL framework was developed to make the adequacy of the constituent sequences explicit and auditable, and a pathway built on uninterpretable images cannot deliver the detection rates reported at expert centers [38,39]. The corollary for any unit comparing biopsy routes is that the comparison is partly moot until MRI quality is assured, and that investment in acquisition protocols, reader training, and quality auditing—increasingly supported by artificial-intelligence tools [77]—yields returns that are, in a sense, prior to and larger than those from optimizing the biopsy route alone.

## 12. Conclusions

The contemporary transperineal MRI-targeted fusion pathway and the historical transrectal systematic pathway are, in aggregate, comparably effective at detecting clinically significant prostate cancer, but they are not interchangeable. The transperineal route distinguishes itself most clearly through a substantial reduction in infectious complications and rectal bleeding, through superior sampling of anterior and apical disease, and through favorable cost-effectiveness and antimicrobial stewardship; it asks of the patient a somewhat more uncomfortable procedure, the discomfort of which can be mitigated by anesthetic technique. Rather than a universal mandate, the evidence supports an individualized decision in which infection-risk reduction and lesion location are the dominant considerations, with MRI quality and PI-RADS interpretation underpinning the success of any targeted approach regardless of route. For most biopsy-naive and prior-negative men in routine practice, these considerations will favor a transperineal MRI-targeted approach; for men with exclusively posterior disease, with limited access to transperineal expertise, or with particular sensitivity to procedural discomfort, an optimally prophylaxed transrectal MRI-targeted approach remains a reasonable and evidence-consistent choice.

What should not be lost in the route-versus-route framing is how much the two pathways now share. Both are, in their contemporary forms, MRI-directed; both sample identified targets rather than relying solely on blind templates; and both have benefited from the same decade of evidence—and the same emerging adjuncts, from biomarkers to micro-ultrasound to artificial intelligence—that has made prostate cancer diagnosis more accurate and less harmful than it was. The migration from transrectal systematic biopsy to transperineal MRI-targeted biopsy is best understood not as the replacement of a bad test by a good one but as the maturation of a diagnostic pathway in which imaging increasingly does the work of localization, and the biopsy is reduced to the precise, safe acquisition of tissue from a known target. The remaining differences between the routes—real and clinically meaningful as they are for infection, for anterior disease, and for patient comfort—are refinements within a shared and substantially improved paradigm, and the task for the clinician is to apply those refinements thoughtfully to the patient in front of them rather than to adjudicate a contest between techniques.

## Figures and Tables

**Table 1:** Principal comparative studies and syntheses of transperineal versus transrectal biopsy and of MRI-targeted versus systematic sampling discussed in this review.

Study	Design	Comparison	Principal Finding	Ref.
*Targeting-axis studies (background—both arms transrectal; MRI-targeted vs. systematic sampling)*				
PROMIS	Paired cohort	mpMRI vs. TRUS systematic	MRI sensitivity 93% vs. 48% for csPCa; ~27% could avoid biopsy	[18]
PRECISION	RCT	MRI-targeted vs. TRUS systematic	csPCa 38% vs. 26%; fewer insignificant cancers (9% vs. 22%)	[11]
MRI-FIRST	Paired diagnostic	Targeted vs. systematic	Each misses a fraction the other detects; combination best	[19]
STHLM3-MRI	Screening RCT	MRI-targeted vs. systematic	Maintained csPCa detection; ~two-thirds fewer insignificant cancers (4% vs. 12%)	[21]
Siddiqui/trio	Comparative/RCT	Fusion-targeted vs. systematic	Targeting raises high-grade, lowers low-grade detection	[22,23]
PRECISE	RCT	MRI-targeted vs. systematic	Non-inferior; fewer cores and fewer insignificant cancers	[16]
*Route-axis studies (primary comparative evidence—transperineal vs. transrectal)*				
PREVENT	RCT	TP (no abx) vs. TR (targeted abx)	csPCa 53% vs. 50%; lower infection in TP arm	[14]
ProBE-PC	RCT (single-center)	TP vs. TR (same platform)	GG ≥ 2 43.2% vs. 47.1% (NS); no sepsis either arm	[42,43]
PERFECT	RCT (non-inferiority)	TP vs. TR targeted cores	csPCa 47.2% vs. 54.2%; TP non-inferiority not met	[44]
TRANSLATE	RCT (multicenter)	LATP vs. TRUS (full pathway)	GG ≥ 2 60% vs. 54% (OR 1.32, 95% CI 1.03–1.70)	[15]
Uleri	Meta-analysis	TP vs. TR MRI-targeted	csPCa OR 1.11 (95% CI 0.98–1.25); NS	[40]
Zattoni (2024)	Meta-analysis	TP vs. TR (prospective)	csPCa OR 0.9; insignificant OR 1.1; both NS	[41]
Wu	Meta-analysis	TP vs. TR MRI-targeted	Per-patient csPCa RR 1.37; more anterior csPCa	[45]
Diamand	PSM cohort	TP vs. TR MRI-targeted	GG ≥ 2 51% vs. 45% (OR 1.37, 95% CI 1.15–1.63)	[46]
Zattoni (2022)	Meta-analysis	TP vs. TR MRI-targeted	Higher TP detection in apex, anterior, transition zones	[47]

csPCa = clinically significant prostate cancer (ISUP grade group ≥ 2); GG = grade group; RCT = randomized controlled trial; OR = odds ratio; RR = relative risk; CI = confidence interval; NS = not significant.

**Table 2:** Comparative harm and patient experience profile of transperineal (TP) versus transrectal (TR) prostate biopsy, with representative pooled or randomized estimates.

Outcome	Representative Evidence	Interpretation/Favored Route	Ref.
Sepsis/severe infection	Lower with TP in pooled analyses; 0 sepsis events in TP arms of NORAPP and PREVENT (PREVENT infection difference *p* = 0.059).	Favors TP; enables reduced/omitted prophylaxis.	[14,55,57]
Any infection/febrile UTI	Significantly lower with TP in meta-analysis.	Favors TP.	[45,55]
Rectal bleeding	Substantially lower with TP (no rectal traversal).	Strongly favors TP.	[9,58]
Hematuria/hematospermia	No significant route difference in most analyses.	Broadly equivalent.	[9,58]
Acute urinary retention	Conflicting across analyses; NS in RCT-only data.	Unresolved; likely core-number dependent.	[9,59]
Procedural pain	More frequent moderate/severe immediate pain with TP (OR 1.84).	Favors TR (modifiable by technique).	[15,60]
Erectile function	Transient decline after either route; recovers by ~3–6 months.	Equivalent; not route-specific.	[61]

OR = odds ratio; NS = not significant.

## Data Availability

The original contributions presented in this study are included in the article. Further inquiries can be directed to the corresponding authors.

## Abbreviations

The following abbreviations are used in this manuscript:

ADC	Apparent diffusion coefficient
AI	Artificial intelligence
CI	Confidence interval
csPCa	Clinically significant prostate cancer (ISUP GG ≥ 2, unless otherwise specified)
DCE	Dynamic contrast-enhanced imaging
DWI	Diffusion-weighted imaging
GG	Grade group
ISUP	International Society of Urological Pathology
LATP	Local-anesthetic transperineal biopsy
mpMRI	Multiparametric magnetic resonance imaging
MRI	Magnetic resonance imaging
NS	Not significant
OR	Odds ratio
PI-QUAL	Prostate Imaging Quality
PI-RADS	Prostate Imaging Reporting and Data System
PSA	Prostate-specific antigen
RCT	Randomized controlled trial
RR	Relative risk
T2W	T2-weighted imaging
TP	Transperineal
TR	Transrectal
TRUS	Transrectal ultrasound
UK	United Kingdom
